# Stressed Liver and Muscle Call on Adipocytes with FGF21

**DOI:** 10.3389/fendo.2013.00194

**Published:** 2013-12-18

**Authors:** Yongde Luo, Wallace L. McKeehan

**Affiliations:** ^1^IBT Proteomics and Nanotechnology Laboratory, Center for Cancer and Stem Cell Biology, Institute of Biosciences and Technology, Texas A&M Health Science Center, Houston, TX, USA

**Keywords:** diabetes, FGF21, adipose FGFR1, obesity, stress response

## Abstract

Fibroblast growth factor 21 (FGF21) is an emerging regulator of local and systemic metabolic homeostasis. Treatment with pharmacological levels of FGF21 alleviates obesity and associated metabolic diseases including diabetes. However, beyond anti-obesogenic effects, the normal roles and underlying mechanisms of FGF21 as an endocrine hormone remain unclear. A recent wave of studies has revealed that FGF21 is a stress-induced endocrine factor in liver, muscle, and other tissues that targets adipose tissue and adipocytes through the FGFR1-betaKlotho complex. Adipose tissues and adipocytes within diverse tissues respond with metabolites and adipokine signals that affect functions of body tissues systemically and cells within the local microenvironment adjacent to adipocytes. Normally this is to prevent impaired tissue-specific function and damage to diverse tissues secreting FGF21 in response to chronic stress. Therefore, diverse stressed tissues and the adipose tissue and adipocytes constitute a beneficial endocrine and paracrine communication network through FGF21. Here we attempt to unify these developments with beneficial pharmacological effects of FGF21 on obesity in respect to inter-organ stress communication and mechanisms.

## Introduction

### Endocrine FGFS and the role of FGF21

Since the debut in 2005 (1), fibroblast growth factor 21 (FGF21) has been of growing interest due to its dramatic beneficial effects at pharmacological levels on weight reduction and alleviation of obesity, diabetes, and fatty liver disease (2, 3). These effects include promoting clearance of systemic glucose and lipids (cholesterol, fatty acids, and triacylglycerides) and enhancing insulin sensitivity, adiponectin action, mitochondrial function, thermogenesis, and energy expenditure (1, 2, 4). Unlike most of the FGF family members that require extracellular heparan sulfate proteoglycan and act locally via autocrine and paracrine mechanisms, FGF21 as a member of the endocrine FGF19 subfamily travels through the circulation to sites distal from its origin and acts predominantly as an endocrine hormone (5). Instead of heparan sulfate proteoglycans, the activity of FGF21 is determined by a transmembrane co-receptor betaKlotho (KLB) that is present as a binary complex with FGF receptor tyrosine kinases (FGFR) in several endocrine and metabolic tissues. This allows selective action of circulating FGF21 on the target tissues expressing FGFR1-KLB complex without affecting those expressing FGFR1 alone.

Basal serum levels of FGF21 vary widely among individuals and are generally low in pure strains of laboratory mice (6). Deficiency or overexpression of FGF21 does elicit alterations in metabolic gene expression in the liver and adipose tissue, but without dramatic metabolic phenotypes under normal physiological conditions (7, 8). In contrast, pharmacologic applications of FGF21 in obese and diabetic animals suggest a wide range of effects across multiple tissues with marked anti-obesogenic and anti-diabetic efficacy (1, 2, 4, 9). However, beyond such drastic pharmacological effects in the obese, the possible functions of FGF21 as a circulating hormone in organ-specific physiopathologies have remained elusive.

### FGF21 as an inducible stress hormone from multiple tissues

Studies in mice indicate that FGF21 is inducible and the liver is a major source of circulating FGF21 (9, 10). Parallel to serum levels, expression of hepatic FGF21 under normal physiological conditions is low; however, during prolonged fasting and starvation, both hepatic and serum FGF21 become dramatically elevated. This triggered early proposals that the physiological role of FGF21 was a starvation hormone that regulates ketogenesis essential for brain function during severe carbohydrate deficits (9, 10). A growing number of studies with animals and patients indicate that FGF21 is induced, in addition to starvation and obesity, by diverse pathogenic conditions such as liver injury, viral infection, chemical insult, specific nutritional deficiency, the hepatic regenerative response as well as liver diseases as hepatosteatosis, steatohepatitis, cirrhosis, and liver cancer (11–17). The common feature of all these conditions is that they impose stress on the liver that compromises its role in maintenance of organism metabolic homeostasis. Therefore, hepatic FGF21 appears to be an inducible hepatic stress signal.

In contrast to the liver that is a switching station for processing metabolites in support of other organs and tissues and the adipose tissue that serves as a storage depot of energy and metabolic precursor of lipids, muscle is a major fuel consumer. To meet high aerobic catabolic demands for ATP, muscle secretes myokines that call on liver and adipose tissue to supply adequate fuel. Consistent with this concept, several recent studies indicate that FGF21 is induced in skeletal, heart, and gastrocnemius muscle under conditions that cause local and systemic metabolic stress (18–25). These include patients with a mitochondrial respiratory chain deficiency in myocytes and mice defective in muscular autophagy/mitophagy (20, 26, 27). FGF21 may be an insulin and AKT-regulated myokine and its expression is associated with chronic muscular hyperinsulinemia or lipodystrophy (22, 23). Increases in muscle contraction such as in chronic exercise also upregulates muscular FGF21 production (21, 25). Fe-S cluster-deficient muscles in patients showed a dramatic upregulation of FGF21 expression and elevated levels of circulating FGF21 (20). This indicates that muscle stressed by perturbations in mitochondrial energy metabolism responds by increasing the secretion of FGF21 into the circulation.

In addition to liver and muscle, it has been suggested that FGF21 is inducible and secreted into the circulation in other tissues and organs under stress conditions. Brown adipose tissue (BAT) may be a source of FGF21 in response to cold stress and fetal-to-neonatal transition (28, 29). White adipose tissue (WAT) has been reported to secrete FGF21 under some conditions (30). However, a physiological contribution of stress-induced adipocyte FGF21 to systemic FGF21, or an autocrine activity of FGF21 within WAT, BAT, and adipocytes in the local microenvironment, remains to be validated. Since stressed pancreas has been reported to be a beneficiary of external FGF21 (31, 32), it is also a candidate for induction of FGF21 as a signal for aid from adipose tissue.

These studies suggests that the activation of FGF21 in liver, muscle, and other tissues is a response of these tissues and organs to stress triggered by external challenge and internal cellular pathogenesis that lead to defects in metabolic homeostasis. In support of this notion, several nuclear receptors and transcription factors that are involved in metabolic stress responses regulate FGF21 expression. These include PPARα/γ, RAR, ChREBP, SREBP1c, LXR, STAT3/5, p53, and ATF4 (9, 16, 26, 33–39). The question is why these organs when under stress use FGF21 as a systemic alarm signal.

### Adipocytes are the target of FGF21 from multiple tissues

Several recent studies using direct ablation of FGFR1 or KLB in adipocytes, administration of activating antibody targeting specifically the FGFR1-KLB complex, or induction of dysfunctional adipose tissue (40–44) provide compelling evidence that adipose tissue and adipocyte FGFR1-KLB is the primary tissue and molecular target of inter-organ messenger FGF21, respectively. Although many types of tissues and cells express FGFR1 alone, mature adipocytes co-express FGFR1 with KLB, the transmembrane co-receptor for FGF21 and FGF19 (45). Similar to the general ablation of FGF21 (8), adipocyte-specific FGFR1 ablation resulted in few overt changes in adipogenesis, adipose secretory function, and systemic metabolic status under normal physiological conditions (46). However, under the metabolic stress of starvation, the absence of adipocyte FGFR1 caused increased adipocyte lipolysis. This occurred concurrently with elevation of serum triglycerides and fatty acids while indirectly causing an increase in hepatic lipogenesis and steatosis (46). This indicated that under such conditions adipocyte FGFR1 signaling dampens adipocyte lipolysis while concurrently dampening hepatic steatosis. Paradoxically yet most dramatically, the adipocyte FGFR1 deficiency abrogated nearly all the anti-obese and anti-diabetic effects elicited by pharmacological levels of FGF21 (40, 43). The alleviation of muscular abnormalities caused by muscular mitochondrial energy dysfunction and defects in autophagy/mitophagy appear to be in large part due to effects of induced FGF21 on adipose tissue (26, 27). Adaptation to cold stress may be aided by elevated levels of FGF21 that acts on BAT to promote pro-thermogenic mitochondrial activity (28, 29).

**Figure 1:**
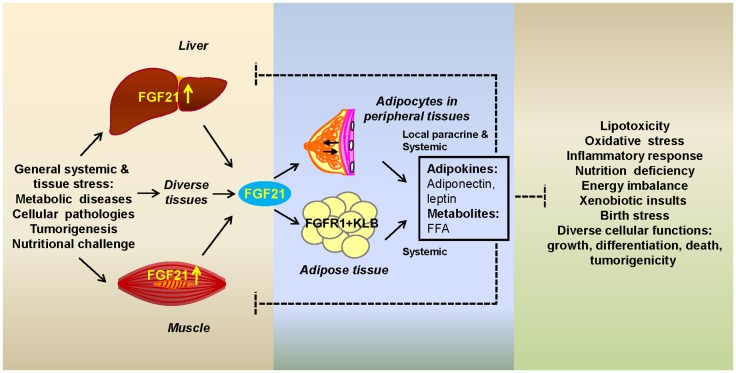
**Fibroblast growth factor 21 as a stress hormone acting via a tissue-adipocyte communication axis**. Stressed tissues through induction of FGF21 (left box) alert adipocytes by activating the adipocyte FGFR1-KLB complex (center box). Adipocytes respond with secretory metabolic products and adipokines that alleviate tissue-specific stress through modification of cellular pathways whose alterations (right box) are a potentially destructive result of systemic or internal cellular stress (left box). Systemic FGF21 acts on adipocytes in major adipose depots or in the microenvironment of peripheral tissues. Breast is shown as an example of the latter. FGF21-induced adipocyte secretory products, such as adiponectin, leptin, and free fatty acids (FFAs), act both systemically or in the local microenvironment (center box) to affect stressed organs or parenchymal cells, respectively.

Taken together, these studies indicate that stress messenger FGF21 and adipose FGFR1-KLB team up to respond to stress caused by a wide range of metabolic abnormalities and pathogenesis. The stress-responsive communication mediated by extra-adipose FGF21 and adipocyte FGFR1-KLB occurs predominantly between adipose tissue and at least two tissues whose roles in metabolism are quite different, but when stressed have major consequences on the organism: (1) liver whose role is to maintain lipid, carbohydrate, and organism metabolic homeostasis through synthesis, degradation, and temporary storage; and (2) muscle which is a major fuel consumer predominantly powered by the oxidation of fats and carbohydrates (Figure 1). Although so far liver and muscle have received the most attention as two major organs that send out FGF21 as a stress signal, other organs may utilize the same mechanism to call on adipocytes in major fat depots (WAT and BAT) or in the microenvironment of diverse tissues. Within the microenvironment of tissues that contains ectopic or interspersed adipose tissue or adipocytes, such as the breast, bone marrow, and the perirenal and epicardial regions, FGF21 may serve to modify adipocyte signaling that affects parenchymal cell functions through a paracrine mechanism. Within stressed adipose tissues which are predominantly adipocytes, both paracrine and autocrine mechanisms may be at play. The ultimate effect of the communication initiated by the FGF21 messenger is to elicit beneficial signals from adipocytes that aid in reduction of the effects of stress and prevention of tissue-specific damage and pathologies resulting from prolonged stress.

### The role of adipose tissue in the FGF21 stress response

As a first responder to the SOS message carried by FGF21, how do adipocytes through FGF21-mediated activation of FGFR1-KLB aid stressed tissues and in particular liver and muscle? In addition to as a reservoir of energy, adipose tissue is an endocrine organ capable of systemic signaling through its lipid metabolites and adipokines (47, 48). A number of effects of FGF21 on adipose tissue adipocytes have been reported. These include the induction of expression of nuclear receptors and coactivators, diverse enzymes and their cytoplasmic regulators involved in pathways for glucose, lipid, and energy metabolism, and the broad changes in systemic and local tissue metabolic substrates and control pathways (1, 2, 4, 9, 40, 43).

Two major effects of FGF21 directly on adipose tissue stand out that may contribute to relief of metabolic stress on liver and muscle. Activation of the adipocyte FGFR1-KLB complex modulates the rate of adipocyte lipolysis and fatty acid oxidation that indirectly relieves hepatocyte steatosis or muscular lipid overload. Upregulation of expression of genes involved in fatty acid oxidation, browning of white fat and thermogenesis is also thought to collectively contribute to the anti-obesogenic effects (1, 29, 40, 49) in the obese. In contrast, activation of the adipocyte FGFR1-KLB complex during starvation dampens the rate of adipocyte lipolysis, which may help to extend energy usage. Secondly, recent studies show that FGF21 regulates the endocrine function of adipocytes including the secretion of adipokines that act on diverse tissues throughout the body. Notably the adipokine adiponectin is upregulated and leptin downregulated by FGF21. They appear to mediate many of the systemic and local effects of FGF21 (50, 51). Reduction of effects of a lipotoxic environment and oxidative stress is a common feature of adiponectin in liver, muscle, heart, kidney, pancreas, and endothelium (52). Thus, both the regulation of fatty acid metabolism and modifications of adipokine profile by FGF21 are favorable for the relief of liver’s steatotic burden that, when extreme and chronic, causes irreversible hepatic damage (Figure 1). Similarly, these FGF21-induced adipocyte responses are favorable for reduction of lipid overload and lipotoxicity in stressed or diseased muscle and possibly other tissues when autophagy/mitophagy, the mitochondria-ER-Golgi network or homeostasis in insulin function and glucose, lipid, and energy metabolic networks is compromised (34, 53).

Are adipocytes via the FGFR1-KLB partnership the primary or even specific target of systemic or local FGF21 signal observed in diverse body tissues? FGF21-stimulated changes in systemic metabolites and adipokines whose receptors are distributed widely have potential to indirectly affect practically all body tissues. Moreover, adipocytes that are characterized by expression of FGFR1-KLB and present in the microenvironment of most, if not all, tissues have potential to affect functions of adjacent tissues via FGF21-controlled paracrine products in the microenvironment. A careful dissection of both FGFR isotypes and KLB and signals elicited by the partnership in parenchymal and adipocytes of diverse organs that show a response to systemic FGF21 is required to clarify whether non-adipocyte cells constitute additional direct targets for FGF21. It has been suggested that FGF21 may signal directly in hepatocytes (54), pancreatic islet beta cells (31, 32), and cells in the suprachiasmatic nucleus of the hypothalamus and the dorsal vagal complex of the hindbrain (55, 56).

## Conclusion and Future Perspective

Since the discovery of the regulatory function of FGF21 in metabolism in 2005, studies have focused on the dramatic systemic pharmacological effects and clinical potential of FGF21 in alleviation of obesity and diabetes. These have obscured its general role as a stress hormone in organ-specific physiopathologies with adipose tissue as its tissue target and adipocyte FGFR1-KLB as its molecular target. The adipocyte FGFR1-KLB complex accounts for the weight reduction and anti-diabetic effects of pharmacological FGF21 (40–44). It also likely accounts for the stress-reducing metabolic effects of FGF21 in tissues undergoing a wide range of conditions causing systemic metabolic as well as local tissue and cellular stress. Induced FGF21 expression may be of utility as a biomarker for these conditions. In addition to obesity and its associated metabolic syndromes, pharmacological levels of FGF21 may be of therapeutic benefit for chronic tissue-specific stress-related diseases.

One important question is whether the treatment of metabolic abnormalities accompanying obesity and diabetes and the relief of stress in diverse tissues occur through the same or overlapping adipocyte signals controlled by FGF21 (Table 1). The uncoupling of lipolysis and lipogenesis by FGF21-FGFR1-KLB directly in adipocytes and indirectly in hepatocytes together with FGF21-stimulated “browning” of fat and thermogenesis have been suggested to create a state of futile cycling and enhanced energy expenditure that causes overall reduction of body weight and systemic lipids in the obese (1, 29, 40, 46, 49). Under the life-threatening stress of prolonged starvation, this same FGF21-FGFR1-KLB mediated uncoupling of overall lipid and carbohydrate metabolism may serve to stretch out lipid reserves for brain fuels to preserve consciousness and reduce neural stress for as long as possible until feeding resumes (9, 46). FGF21-elicited adipocyte signals appear to commonly alleviate the extent of hepatic steatosis under the opposite metabolic extremes of obesity and starvation, and the muscular metabolic stress associated with autophagic defects and mitochondrial respiratory dysfunction.

**Table 1:** **Outstanding questions**.

How do different stress conditions commonly induce FGF21 expression?
Is FGF21 of biomarker value for tissue stress?
Do the roles of FGF21 in the stress response and treatment of obesity occur through the same mechanisms?
Do tissues/organs other than liver and muscle also utilize FGF21 as a stress signal?
What is the full spectrum of FGF21-controlled adipocyte signals (metabolites and adipokines) that contribute to alleviation of stress?
How does FGF21 direct KLB-FGFR1 kinase signaling to control adipose tissue function instead of mitogenesis?

Adipokines have emerged as FGF21-induced endocrine signals that also communicate back to stressed tissues. The upregulation of adiponectin currently is in the spotlight (40, 50, 51) although the role of the full spectrum of FGF21-controlled adipokines deserves investigation (Table 1). The many facets of the roles of FGF21 as a stress hormone that recruits the aid of adipose tissue adipocytes and its ramifications for stress-related diseases promise to be a broad and fruitful field of future study.

In addition to the tissue-specific responses to FGF21-stimulated adipocyte signals that alter metabolism to reduce tissue stress, the mechanisms that control the induction of FGF21 in specific tissues in crisis also requires clarification (Table 1). Multiple transcription factors including nuclear receptors have been implicated in control of FGF21 expression in different tissues. Some of them are involved in the intrinsic cellular stress-responsive pathways, such as the SREBP1c and ATF4 in the ER stress response that are associated with the Golgi, peroxisome (53, 57), and mitochondria and involved in lipid, energy, and glucose metabolism. Future studies may reveal novel tissue-specific FGF21-mediated stress response pathways and mechanisms.

The overarching canonical role of members of the diverse FGF superfamily in the adult is coordination of tissue homeostasis through autocrine and paracrine growth-promoting signaling controlled by matrix heparan sulfate. With the loss of affinity for heparan sulfate and gain of selectivity for the FGFR1-KLB complex that predominates in mature adipocytes, FGF21 has evolved as an inter-organ stress signal to adipocytes for negative regulation of systemic and cellular metabolic stress. This concept may also apply to evolution of other endocrine FGFs referred to as the FGF19 subfamily. The inter-organ crosstalk between ileal FGF19 and liver FGFR4-KLB is a negative regulatory mechanism of postprandial bile acid levels serving to prevent stress and damage resulting from exposure to prolonged and elevated levels of bile acids (58). Similarly, the negative regulation of mineral re-absorption levels signaled by the crosstalk between bone FGF23 and kidney FGFR1-KL (59) may limit potential stress and damage from mineral overload. The effects of the FGF19 subfamily are pleiotropic and complex on both local tissue and organism levels. Detailed dissection of the action of each endocrine FGF in inter-organ or cross-tissue communication and stress relief promises to be a novel conceptual base for pursuit of complete understanding of the biology of endocrine FGFs.

## Conflict of Interest Statement

The authors declare that the research was conducted in the absence of any commercial or financial relationships that could be construed as a potential conflict of interest.
